# Supplementary material for: Safe drugs with high potential to block malaria transmission revealed by a spleen-mimetic screening
Source: Nat Commun. 2023 Apr 7;14:1951. doi: 10.1038/s41467-023-37359-2 (PMC10082216; doi:10.1038/s41467-023-37359-2)
Supplement: Supplementary file 1 — Supplementary information [file 41467_2023_37359_MOESM1_ESM.pdf]

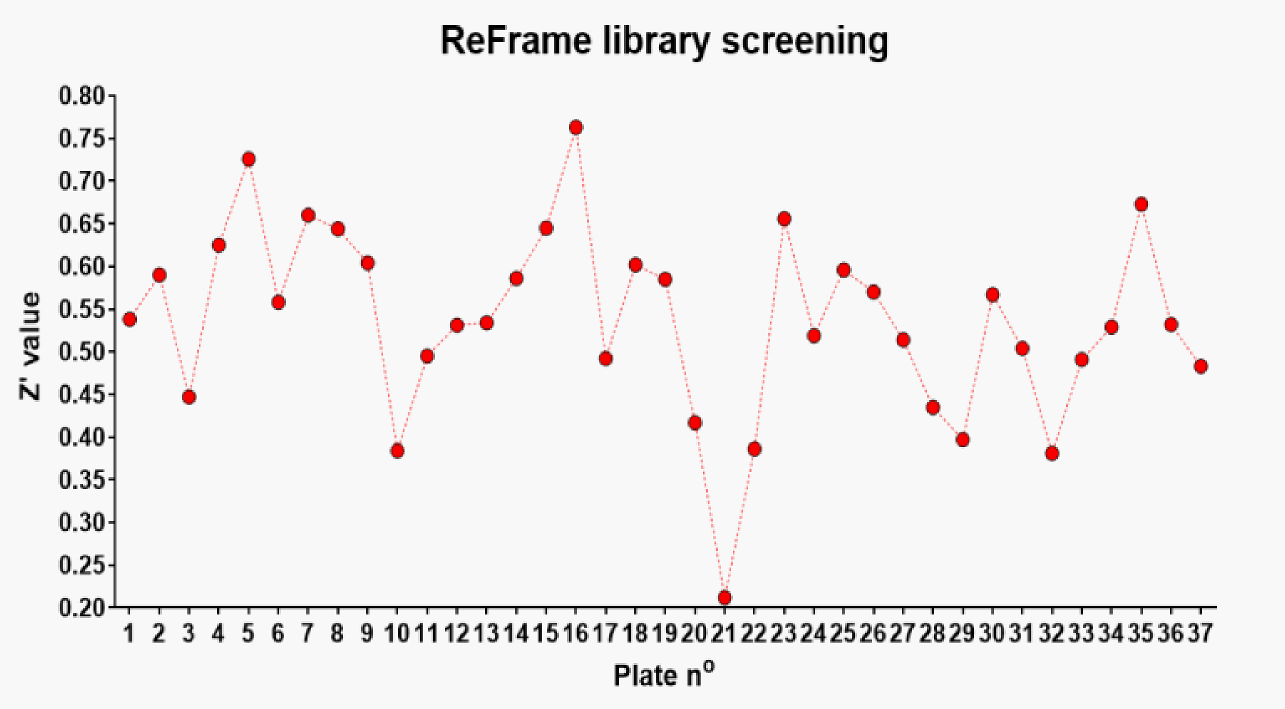

Figure S1. Single plate Z' values of the ReFrame library. For each plate of the ReFrame library, a Z' value was calculated with the formula $Z' = (1 - ((3 * SD_{PC}) + (3 * SD_{NC})) / | RR_{PC} - RR_{NC}|)$, where SD is the standard deviation, PC is the main positive control (NITD609 0.5 µM), NC is the negative control (DMSO) and RR is the main retention rate's value.

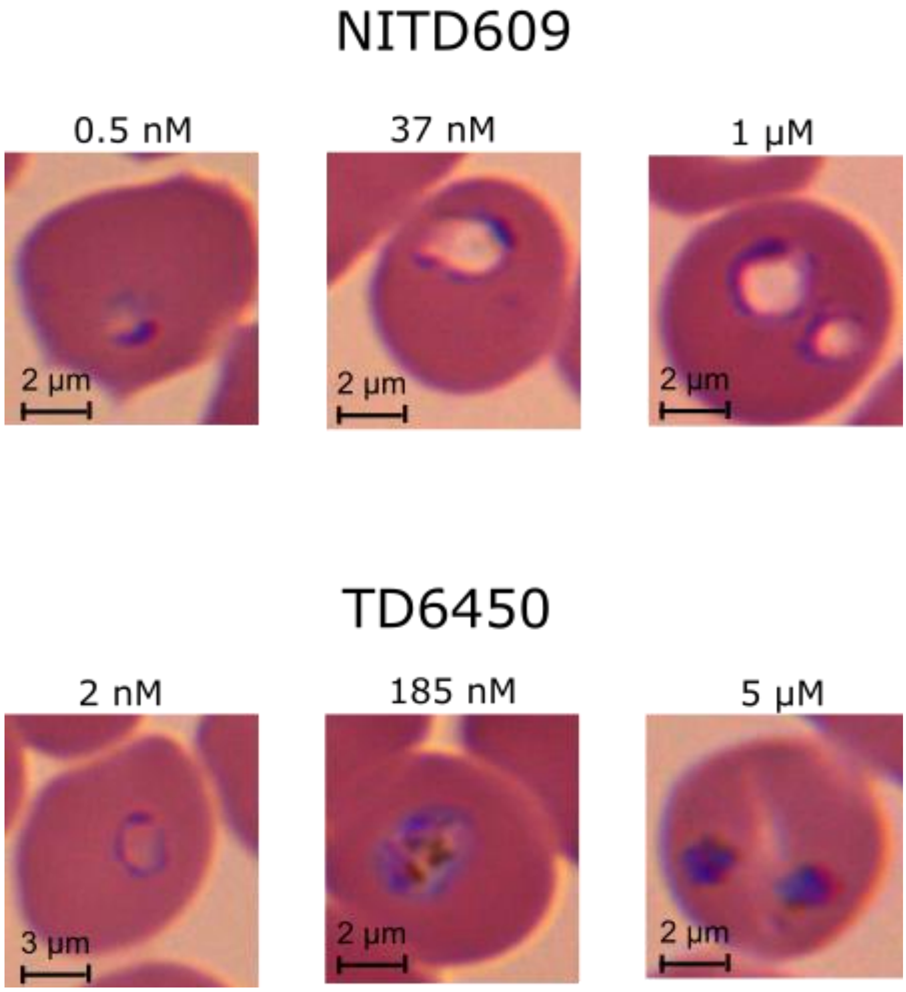

Figure S2. Giemsa-stained images of drug-exposed ring-stage parasites. Giemsa-stained erythrocytes infected by a ring-stage of *P.falciparum* parasite exposed 48 hours to NITD609 (higher panel) and TD-6450 (bottom panel). The optical microscopic observation was repeated 5 times.

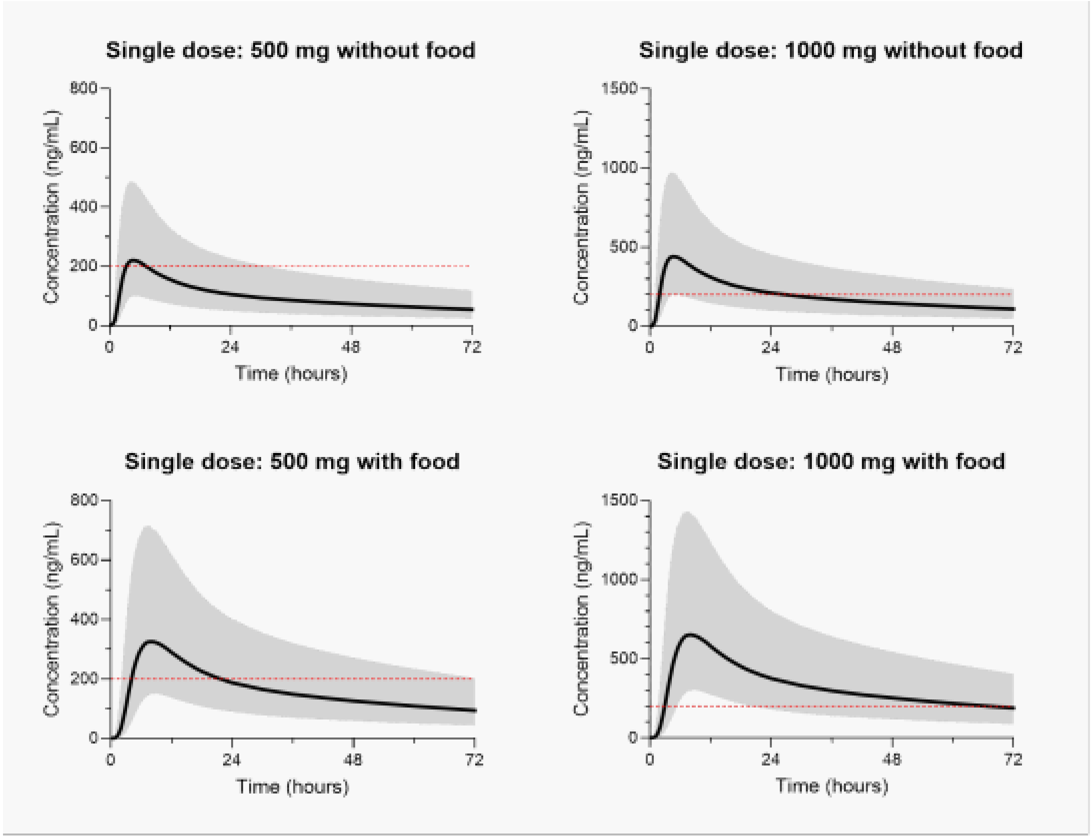
Single dose: 500 mg without food
Single dose: 1000 mg without food
Single dose: 500 mg with food
Single dose: 1000 mg with food
Concentration (ng/mL)
Time (hours)
0
24
48
72
200
400
600
800
500
1000
1500

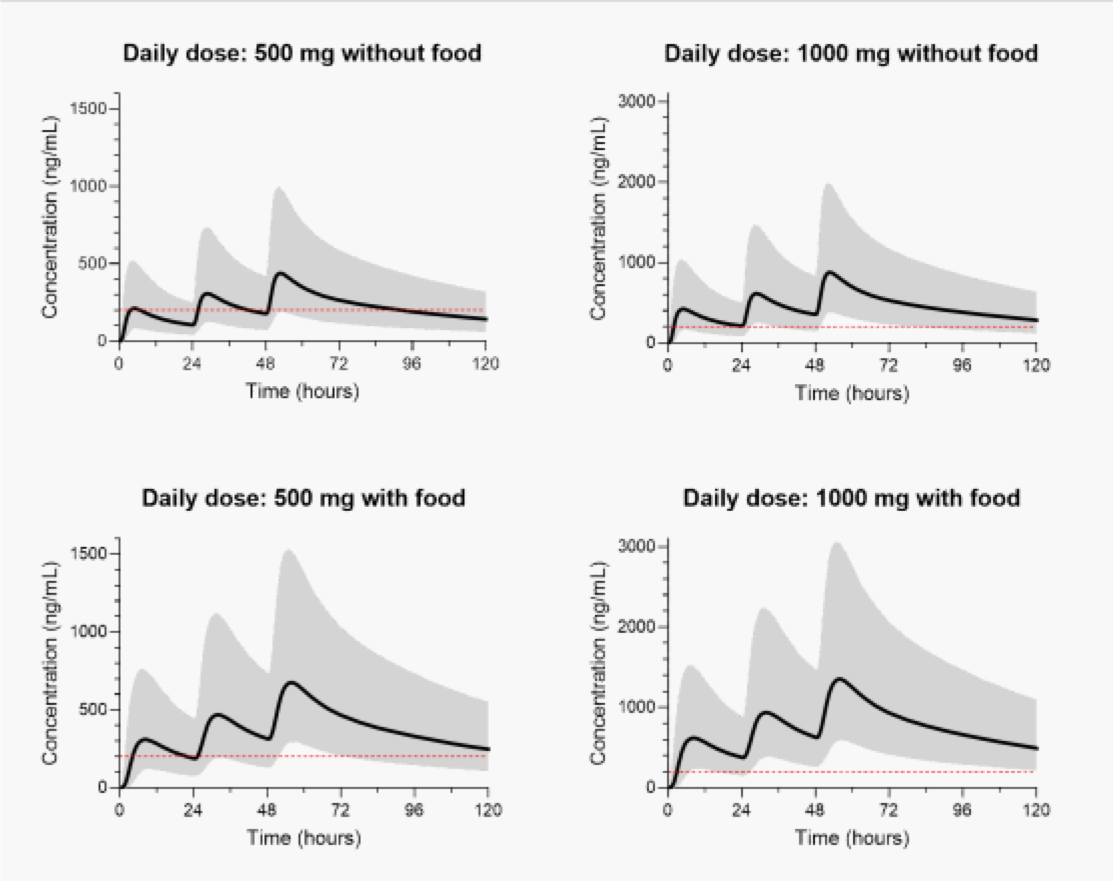
Daily dose: 500 mg without food
Daily dose: 1000 mg without food
Daily dose: 500 mg with food
Daily dose: 1000 mg with food
Concentration (ng/mL)
Time (hours)
0
24
48
72
96
120
500
1000
1500
2000
3000

Figure S3. Mean simulations from the population of TD-6450's PK model (80 kg typical subject). Simulations of concentration times in healthy subjects based on phase I study modelling, when 500 and 1000 mg single (higher panel) and multiple (3 doses once daily, bottom panel) dose is administered, with or without food. Black line is the mean value, the grey shaded area shows the 90% prediction interval. 200 nM concentration is indicated with a red dotted line. Iter-individual variability is shown in grey.

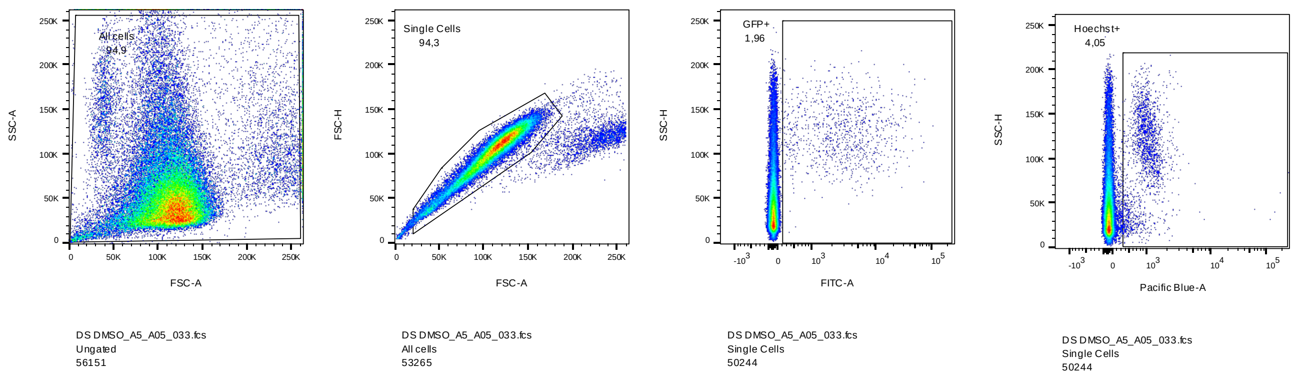

Figure S4. Example of gating strategy for flow cytometry analysis. Post-screening validation microsphiltration's experiment: single downstream sample of DMSO-treated stage gametocytes. From left to right: first plot (FSC-A and SSC-A) and gate to select the cell population; second plot (FSC-A and FSC-H) and gate to exclude doublet; third plot (FITC-A and SSC-H) and gate to select GFP positive cells; fourth plot (Pacific Blue and SSC-H) and gate to select Hoechst positive cells.

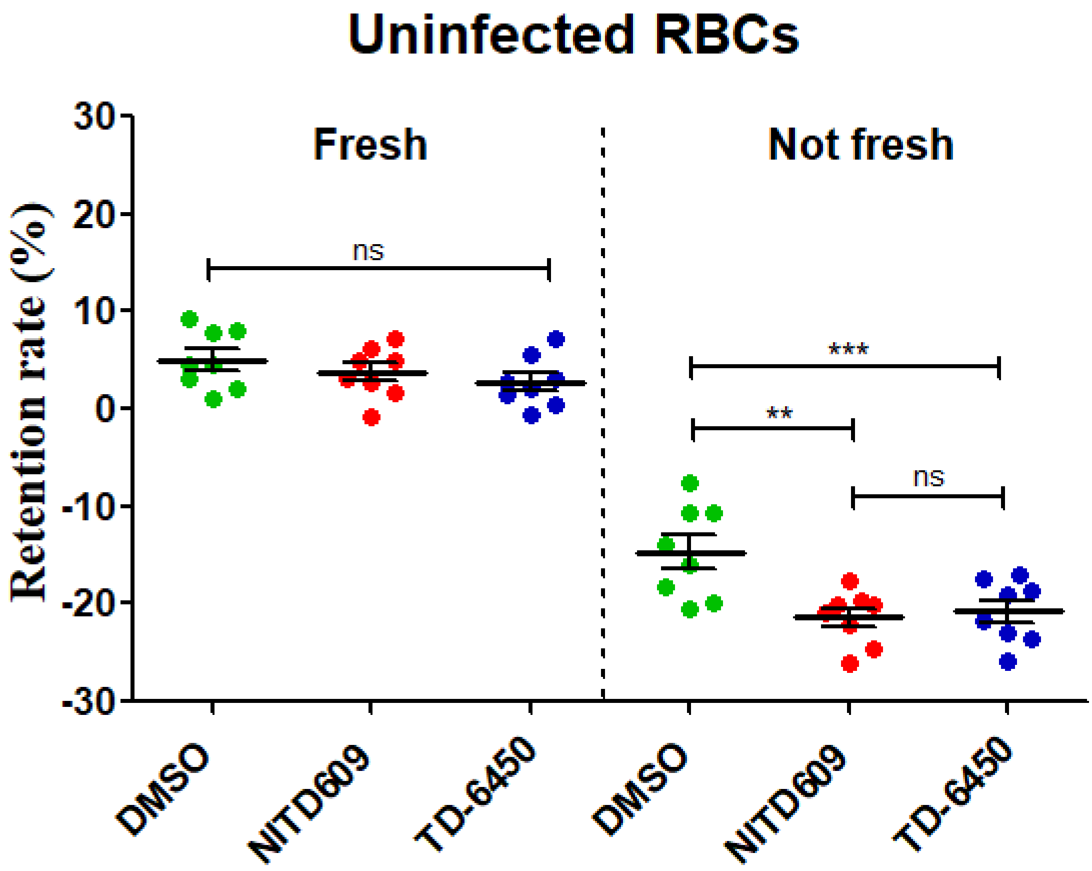

Figure S5. Stiffening effect of selected hits on uninfected RBCs. Retention of uninfected RBC exposed to DMSO, TD-6450 or NITD609 during 24 hours before microsphiltration. RBC were either used less than 72 hours after collection (“Fresh”) or following 2 weeks in culture conditions at 37°C with medium change every other day (“Sham Cultured”). Positive values correspond to retention and negative values to enrichment of the RBC population of interest following filtration. Statistical analysis was performed with one-way Anova test. P values are: DMSO vs NITD609 0.0012 and DMSO vs TD-6450 0.0007. Individual p values legend: *p = 0.05–0.01, ** p = 0.01–0.001, *** p = 0.001–0.0001, **** p < 0.0001. Source data are provided as a Source Data file.

Table S1. ReFrame library hitlist with chemical structures and relative IC_{50} for both killing effect and stiffening activity. Results in previous assays to show both effect in Plasmodium replication (72-hour P.falciparum Dd2 SybrGreen Protein Binding Fold Shift (PBFS) assay) and cytotoxicity (HEK293T and HepG2 72-hour Cytotoxicity). IC50 were shown for these assays when the compound was selected during primary screening.

**Name**	**Chemical structure**	**Killing IC50 (µM)**	**Stiffening IC50 (µM)**	**Group**	**Molecular target**	**"72-h Dd2-SybrGreen PBFS" assay IC50 (µM)**	**Cellular cytotoxicity assays IC50 (µM) (HEK293T & HepG2)**
Atiprimod dimaleate	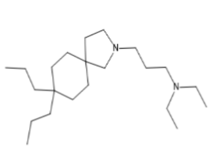	6.67	N/A	Kinase and phosphatase inhibitors	Human PKB/Akt	NC	NC
Decamethoxine	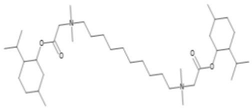	8.1	1	Antibiotics & antivirals	Unknown	0.061	3.3 & 4.3
Oligomycin A	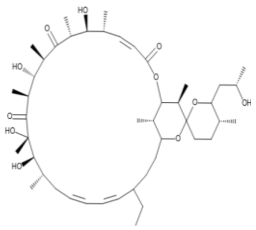	N/A	0.5	Antibiotics & antivirals	Human HIF-1	NC	NC
Acetomeroctol	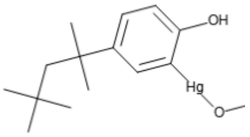	7.2	5	Antibiotics & antivirals	Unknown	NC	0.462 & 1.92
KF 66854	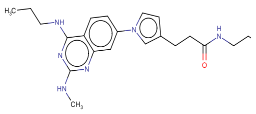	0.76	3.6	Others	5-HT4 receptor	1.64	NC & 5.08
Potassium antimonyl tartrate	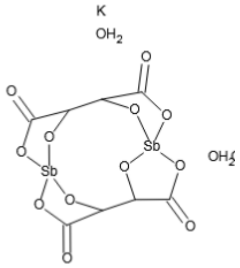	3.63	10.7	Others	Unknown	NC	NC
Bortezomib	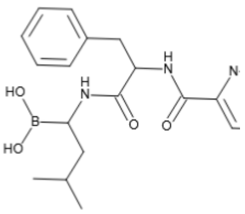	2.06	N/A	Others	Human Proteasome subunit beta type-5 & 1	NC	NC

Ammonium trichlorotellurate	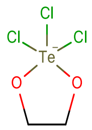	13.7	2.2	Antibiotics & antivirals	Unknown	NC	NC
Methylthioninium chloride (Methylene Blue)	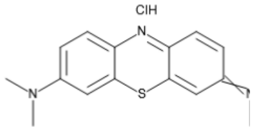	4.85	2.8	MAO inhibitors	Human guanylate cyclase & nitric oxide synthase	NC	NC
Quisinostat	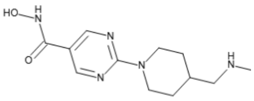	5.2	3.5	Anti-cancer: HDAC inhibitors	Human HDAC	NC	NC
Gramicidin	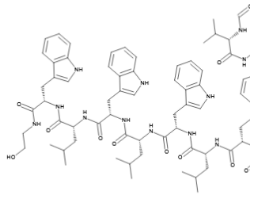	0.008	0.07	Antibiotics & antivirals	Bacterial membranes	NC	NC
Abexinostat	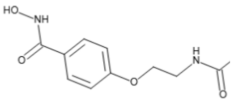	2	0.64	Anti-cancer: HDAC inhibitors	Human HDAC	NC	NC
Eseroline	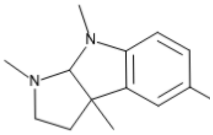	1.565	2.3	Others	Human AcHEIs	NC	NC
CRA-026440	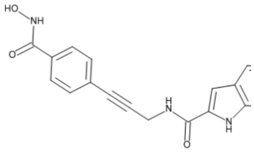	16	0.55	Anti-cancer: HDAC inhibitors	Human HDAC	0.063	0.081 & 0.011
Alexidine dihydrochloride	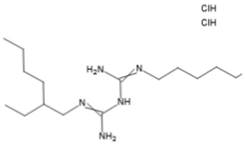	41	0.55	Antibiotics & antivirals	Unknown	0.032	3.33 & 3.06
Leuco methylthioninium salt (Methylene Blue salt)	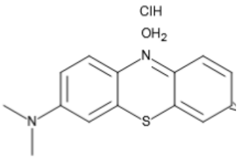	23.25	N/A	MAO inhibitors	Human guanylate cyclase & nitric oxide synthase	NC	NC
N-tert-butylisoquine	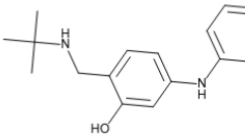	9.55	9.45	Antimalarial agents	Pf Hemoglobin degradation	NC	NC
Unidentified compound	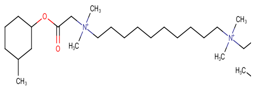	3.3	5.04	Others	Unknown	NC	NC

Romidepsin	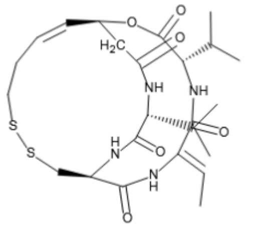	20	5	Anti-cancer: HDAC inhibitors	Human HDAC	NC	NC
Bisantrene HCl	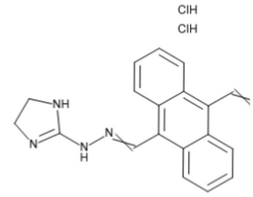	2.2	N/A	Others	Human DNA topoisomerase II	0.065	0.054 & 0.062
Auranofin	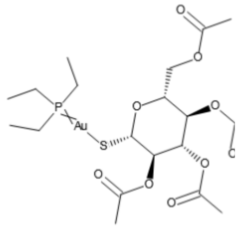	2.575	3.5	Others	Human TrxR	NC	NC
NVP-BGT226	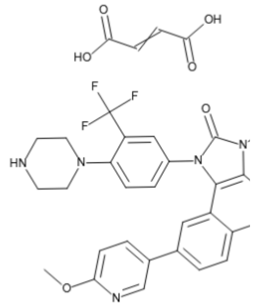	0.013	1.6	Kinase and phosphatase inhibitors	Human PI3K	NC	NC
Oligomycin B	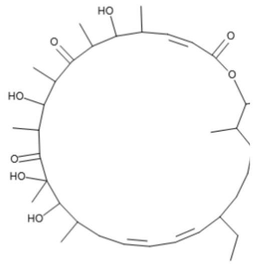	5.8	1.55	Antibiotics & antivirals	Human HIF-1	NC	NC
Homoharringtonine	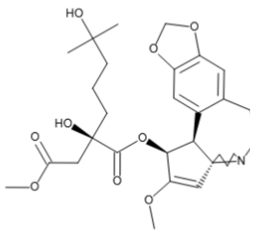	1.73	2.3	Others	Human Stat3	0.007	0.032 & 0.122
DDD498	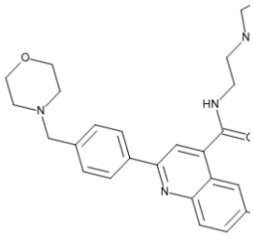	1.7	2.6	Antimalarial agents	Pf EF2	0.004	NC

Unidentified compound	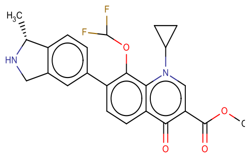	N/A	0.2	Others	Unknown	NC	NC
Unidentified compound	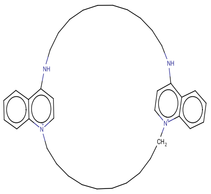	7.45	1.75	Others	Unknown	NC	NC
NITD609	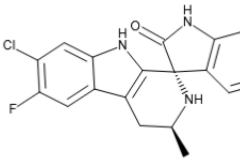	0.15	0.11	Antimalarial agents	Pf ATPase 4	NC	NC
Bismuth ethanedithiol	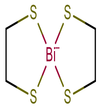	1.3	1.5	Antibiotics & antivirals	Unknown	NC	NC
YM 161514	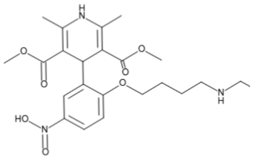	5.55	3.6	Others	Human Beta-1 adrenergic receptor	2.26	NC & 3.97
SR-26050	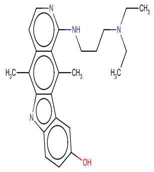	2.6	2.9	Others	Unknown	NC	NC
PPA904	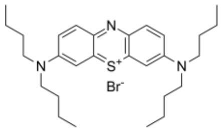	2.415	5.05	Others	Unknown	NC	NC
Tyrothricin	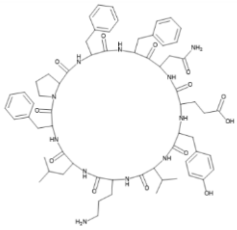	0.29	5.7	Antibiotics & antivirals	Bacterial membranes	0.033	1.8 & 1.9
Pirtenidine	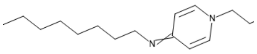	2.785	3.65	Antibiotics & antivirals	Unknown	0.615	1.48 & 0.285
TD-6450	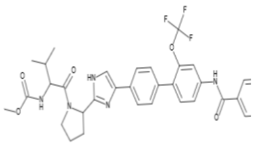	N/A	0.55	Antibiotics & antivirals	HCV NS5A	NC	NC
Cephaeline	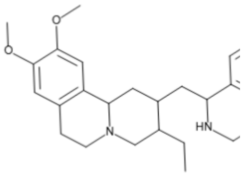	1	3.8	Others	Human 5-HT4 receptor	0.028	0.014 & 0.062

BRD-7929	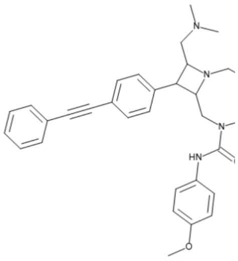	2.2	3.8	Antimalarial agents	Phenylalanil tRNA synthetase	NC	NC
KDU731	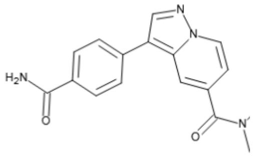	0.09	0.12	Kinase and phosphatase inhibitors	Cp PI4K	NC	NC
VE-822	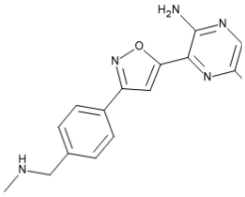	0.71	9.5	Kinase and phosphatase inhibitors	Human ATR kinase	NC	NC
Pyrithione Zinc	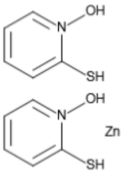	2.5	6.2	Antibiotics & antivirals	Fungal proton pumps	NC	NC
PA92	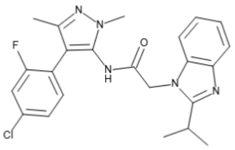	1.15	0.6	Antimalarial agents	Pf ATPase 4	NC	NC
Bispyrithione	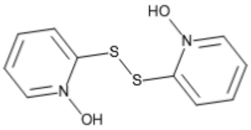	0.92	2.35	Antibiotics & antivirals	Fungal proton pumps	NC	NC
AR-42	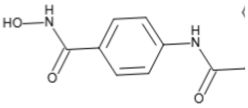	1.5	1.9	Anti-cancer: HDAC inhibitors	Human HDAC	NC	NC
BN-82685	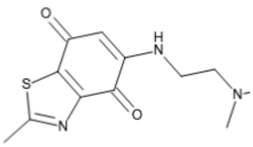	2.8	4.6	Kinase and phosphatase inhibitors	Human CDC25 phosphatase	1.74	2.92 & 1.89
Paranyline	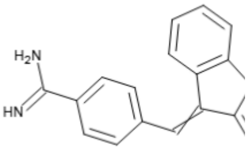	1.9	3.2	Others	Unknown	1.87	1.42 & 0.65
APPCL	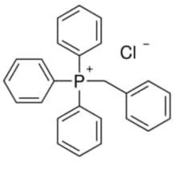	1.2	1.03	Others	Unknown	1.06	0.923 & 0.354

Bruceantin	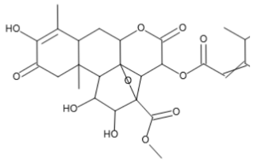	0.16	0.02	Others	Unknown	NC	NC
Sepantronium bromide	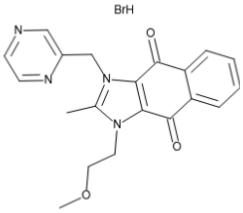	1.35	1.89	Others	Unknown	NC	NC
Ceritinib	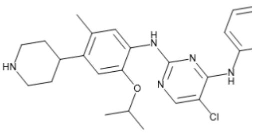	10	10.5	Kinase and phosphatase inhibitors	Human ALK1	NC	NC
Chlorproguanil hydrochloride	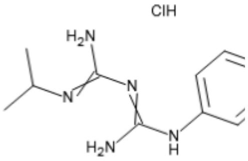	N/A	10.35	Antimalarial agents	Pf antifolate	NC	NC
Daunorubicin	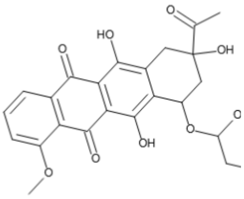	N/A	12	Others	Human topoisomerase I & Iiα	NC	0.039 & 0.126
MMV-390048	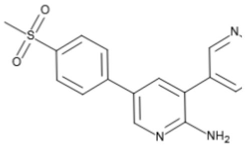	3.86	3.5	Kinase and phosphatase inhibitors	Pf PI4K	0.229	NC
Thimerosal	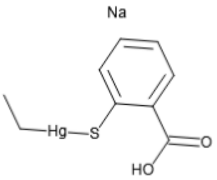	1.5	0.93	Antibiotics & antivirals	Unknown	NC	NC
Peruvoside	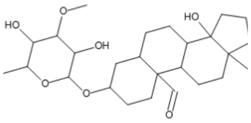	N/A	2.675	Cardiac glycosides	Human ATPase Na^+/K^+ pump	9.95	0.02 & 0.032
Lanatoside A	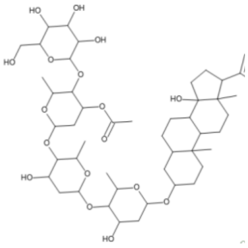	N/A	0.86	Cardiac glycosides	Human ATPase Na^+/K^+ pump	9.95	0.171 & 0.136
Givinostat hydrochloride	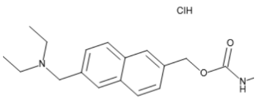	2.1	1.4	Anti-cancer: HDAC inhibitors	Human HDAC	NC	NC

CUDC-907	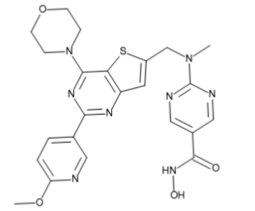	3.32	1.86	Anti-cancer: HDAC inhibitors	Human HDAC	0.018	0.03 & 0.003
MLN 576	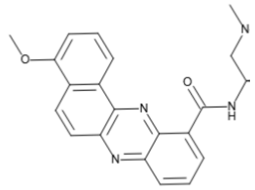	N/A	5.02	Others	Human topoisomerase I & Ii	2.04	0.218 & 0.413
Convallatoxin	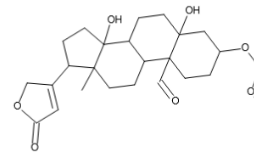	N/A	0.05	Cardiac glycosides	Human ATPase Na^+/K^+ pump	0.016	0.026 & 0.033
Digitoxin	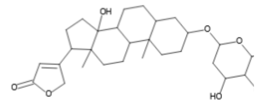	N/A	10.6	Cardiac glycosides	Human ATPase Na^+/K^+ pump	NC	0.008 & 0.011
SkQ1	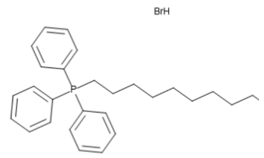	3.85	1.9	Others	Unknown	1.05	0.778 & 3.13
CHR-3996	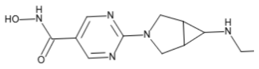	0.07	7.23	Anti-cancer: HDAC inhibitors	Human HDAC	0.015	0.728 & 0.127
Cymarine	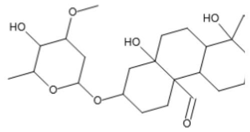	N/A	0.33	Cardiac glycosides	Human ATPase Na^+/K^+ pump	9.95	0.071 & 0.102
Proscillaridin	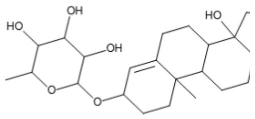	0.3	0.08	Cardiac glycosides	Human ATPase Na^+/K^+ pump	9.95	0.009 & 0.014
Alvespimycin hydrochloride	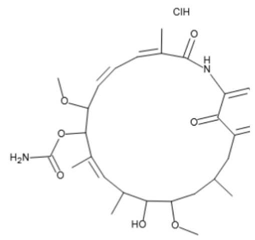	1.75	0.96	Others	Human HSP90	0.272	0.098 & 0.014
Halofuginone	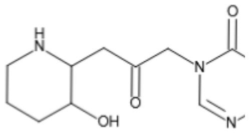	0.24	4.3	Others	Human MMP-2	0.001	0.089 & 0.036
Octenidine	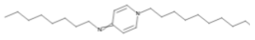	1.25	1.825	Antibiotics & antivirals	Unknown	NC	NC

Panobinostat lactate	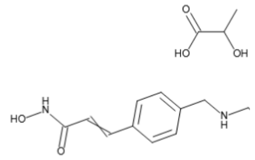	0.3	0.22	Anti-cancer: HDAC inhibitors	Human HDAC	NC	NC
Mitoquinone mesylate	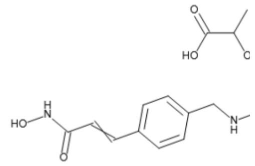	11.7	12.1	Others	Human mitochondria	NC	NC
Stilbazium iodide	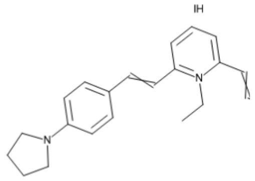	8.745	1.57	Antibiotics & antivirals	Unknown	NC	NC
Zinc Pyrithione	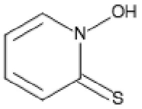	1.675	2.6	Antibiotics & antivirals	Fungal proton pumps	NC	NC
Istaroxime	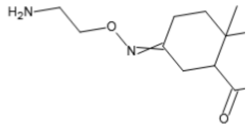	N/A	0.17	Cardiac glycosides	Human ATPase Na^+/K^+ pump	NC	NC
Unidentified compound	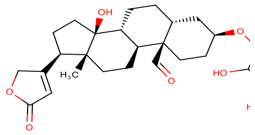	N/A	0.04	Others	Unknown	NC	NC
(S)-(-)-Tetrahydropalmatine (L-THP)	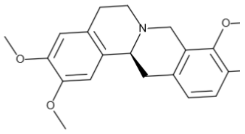	N/A	0.001	Others	Unknown	NC	NC
Myristyl-gamma picolinium chloride	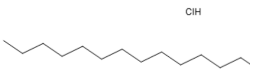	4.15	7.2	Antibiotics & antivirals	Unknown	NC	NC
Narasin	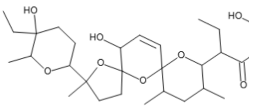	46	43	Antibiotics & antivirals	Dengue virus	NC	NC

Abbreviations:

N/A: not active in dose-response analysis

NC: not captured in primary screening

MAO: mono amino oxidase

HDAC: histone de-acetylase.

Table S2. Kinase Inhibitors and Pathogen Box hit list with chemical structures and relative IC_{50} for both killing effect and stiffening activity.

ID	Chemical structure	Killing IC50 (µM)	Stiffening IC50 (µM)	Library	Molecular target	Main previous screening approaches
GSK1326255A	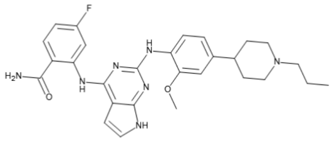	8.4	0.48	Kinase Inhibitors Box	Human IGF1R	*(1–5)*
GSK1173862A	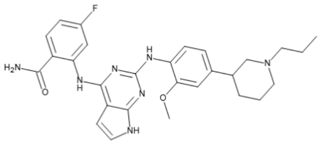	2.4	0.345	Kinase Inhibitors Box	Human IGF1R	*(1, 4–6)*
GSK1220512A	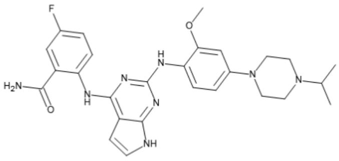	17	2.315	Kinase Inhibitors Box	Human IGF1R	*(1, 5, 7)*
GSK1321730A	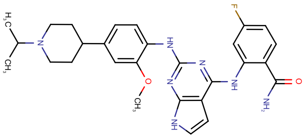	2.44	0.815	Kinase Inhibitors Box[a]	Human IGF1R	Not screened
MMV020081	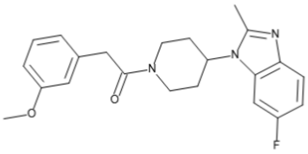	29.3	1.47	Pathogen Box	Pf ATP4	*(8–10)*
MMV667494	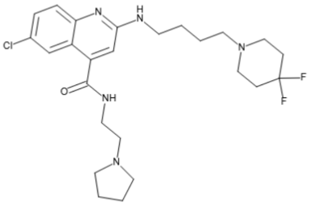	0.07[b]	0.42[b]	Pathogen Box	Pf EF2	*(8–10)*
MMV030734	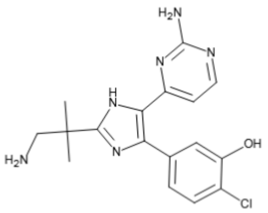	0.21[b]	0.365[b]	Pathogen Box	Pf CDPK1	*(8–10)*

[a]Hits chemical analog not present in the screened library

[b]Specific to female gamete formation

Abbreviations: IGF1R: Insulin-like growth factor 1 receptor

Table S3. Safety and pharmacokinetics in human healthy volunteers of N5SA inhibitors with similar chemical structures compared to TD-6450.

Drug	Chemical structure	Safety	Pharmacokinetics	Intervention	Clinical trial ID
Elbasvir	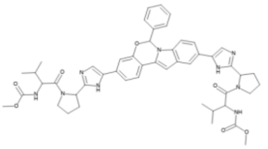	SAE (%): 0<br>AE (%): 37.5	C_{max}: 0.163 µM<br>$t_{1/2}$: 25 hrs	50 mg single dose	NCT01937975
Ledipasvir	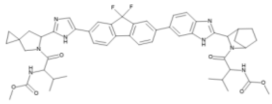	SAE (%): 0<br>AE (%): 42.9	C_{max}: 0.362 µM<br>$t_{1/2}$: 39.9 hrs	90 mg single dose[a]	CTR20160149
Daclatasvir	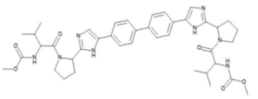	SAE (%): 0<br>AE (%): 16.7	C_{max}: 0.945 µM<br>$t_{1/2}$: 12.4 hrs	30 mg single dose	NCT00859053
Velpatasvir	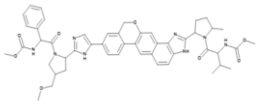	SAE (%): 0<br>AE (%): 42.9	C_{max}: 0.584 µM<br>$t_{1/2}$: 16.8 hrs	100 mg single dose[a]	CTR20160602
Ombitasvir	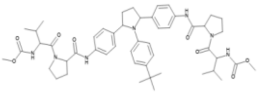	Favorable safety profile	C_{max}: 0.12 µM<br>$t_{1/2}$: 21-25 hrs[b]	25 mg once daily for 14 days[c]	NCT02534870

Abbreviations: SAE: serious adverse event; AE: adverse event

[a]In combination with sofosbuvir 400 mg

[b]Data not taken from this clinical trial *(11)*

[c]In combination with paritaprevir (150 mg) and ritonavir (100 mg)

References:

1. High-throughput screening of a GlaxoSmithKline protein kinase inhibitor set identifies an inhibitor of human cytomegalovirus replication that prevents CREB and histone H3 post-translational modification - PMC (available at https://www.ncbi.nlm.nih.gov/pmc/articles/PMC5817216/).

2. J. M. Elkins, V. Fedele, M. Szklarz, K. R. Abdul Azeez, E. Salah, J. Mikolajczyk, S. Romanov, N. Sepetov, X.-P. Huang, B. L. Roth, A. Al Haj Zen, D. Fourches, E. Muratov, A. Tropsha, J. Morris, B. A. Teicher, M. Kunkel, E. Polley, K. E. Lackey, F. L. Atkinson, J. P. Overington, P. Bamborough, S. Müller, D. J. Price, T. M. Willson, D. H. Drewry, S. Knapp, W. J. Zuercher, Comprehensive characterization of the Published Kinase Inhibitor Set. *Nat. Biotechnol.* **34**, 95–103 (2016).

3. K. T. Homan, K. M. Larimore, J. M. Elkins, M. Szklarz, S. Knapp, J. J. G. Tesmer, Identification and Structure–Function Analysis of Subfamily Selective G Protein-Coupled Receptor Kinase Inhibitors. *ACS Chem. Biol.* **10**, 310–319 (2015).

4. A. Rahman, K. M. Henry, K. D. Herman, A. A. Thompson, H. M. Isles, C. Tulotta, D. Sammut, J. J. Rougeot, N. Khoshaein, A. E. Reese, K. Higgins, C. Tabor, I. Sabroe, W. J. Zuercher, C. O. Savage, A. H. Meijer, M. K. Whyte, D. H. Dockrell, S. A. Renshaw, L. R. Prince, Inhibition of ErbB kinase signalling promotes resolution of neutrophilic inflammation. *eLife* **8**, e50990.

5. D. M. Foulkes, D. P. Byrne, F. P. Bailey, S. Ferries, C. E. Eyers, K. Keeshan, S. Shrestha, W. Yeung, N. Kannan, C. Wells, D. H. Drewry, W. J. Zuercher, P. A. Eyers, Repurposing covalent EGFR/HER2 inhibitors for on-target degradation of human Tribbles 2 (TRIB2) pseudokinase, 305243 (2018).

6. M. D. Matossian, S. Elliott, L. V. Rhodes, E. C. Martin, V. T. Hoang, H. E. Burks, W. J. Zuercher, D. H. Drewry, B. M. Collins-Burow, M. E. Burow, Application of a small molecule inhibitor screen approach to identify CXCR4 downstream signaling pathways that promote a mesenchymal and fulvestrant-resistant phenotype in breast cancer cells. *Oncol. Lett.* **21**, 380 (2021).

7. Application of Computer Modeling to Drug Discovery: Case Study of PRK1 Kinase Inhibitors as Potential Drugs in Prostate Cancer Treatment | IntechOpen (available at https://www.intechopen.com/chapters/55629).

8. J. Calit, I. Dobrescu, X. A. Gaitán, M. H. Borges, M. S. Ramos, R. T. Eastman, D. Y. Bargieri, Screening the Pathogen Box for Molecules Active against Plasmodium Sexual Stages Using a New Nanoluciferase-Based Transgenic Line of P. berghei Identifies Transmission-Blocking Compounds. *Antimicrob. Agents Chemother.* **62** (2018), doi:10.1128/AAC.01053-18.

9. A. S. M. Dennis, J. E. O. Rosling, A. M. Lehane, K. Kirk, Diverse antimalarials from whole-cell phenotypic screens disrupt malaria parasite ion and volume homeostasis. *Sci. Rep.* **8**, 8795 (2018).

10. S. Duffy, V. M. Avery, Identification of inhibitors of Plasmodium falciparum gametocyte development. *Malar. J.* **12**, 408 (2013).

11. D. J. Cada, J. Leonard, T. L. Levien, D. E. Baker, Ombitasvir/Paritaprevir/Ritonavir and Dasabuvir. *Hosp Pharm* **50**, 396–412 (2015).
