# Supplementary material for: Safe drugs with high potential to block malaria transmission revealed by a spleen-mimetic screening
Source: Nat Commun. 2023 Apr 7;14:1951. doi: 10.1038/s41467-023-37359-2 (PMC10082216; doi:10.1038/s41467-023-37359-2)
Supplement: Supplementary file 3 — Reporting Summary [file 41467_2023_37359_MOESM3_ESM.pdf]

nature portfolio

Corresponding author(s): Prof. Pierre Buffet

Last updated by author(s): Jan 21, 2023

Reporting Summary

Nature Portfolio wishes to improve the reproducibility of the work that we publish. This form provides structure for consistency and transparency in reporting. For further information on Nature Portfolio policies, see our Editorial Policies and the Editorial Policy Checklist.

Statistics

For all statistical analyses, confirm that the following items are present in the figure legend, table legend, main text, or Methods section.

n/a	Confirmed	
☐	☒	The exact sample size (*n*) for each experimental group/condition, given as a discrete number and unit of measurement
☐	☒	A statement on whether measurements were taken from distinct samples or whether the same sample was measured repeatedly
☐	☒	The statistical test(s) used AND whether they are one- or two-sided<br>*Only common tests should be described solely by name; describe more complex techniques in the Methods section.*
☐	☒	A description of all covariates tested
☐	☒	A description of any assumptions or corrections, such as tests of normality and adjustment for multiple comparisons
☐	☒	A full description of the statistical parameters including central tendency (e.g. means) or other basic estimates (e.g. regression coefficient) AND variation (e.g. standard deviation) or associated estimates of uncertainty (e.g. confidence intervals)
☐	☒	For null hypothesis testing, the test statistic (e.g. *F*, *t*, *r*) with confidence intervals, effect sizes, degrees of freedom and *P* value noted<br>*Give P values as exact values whenever suitable.*
☐	☒	For Bayesian analysis, information on the choice of priors and Markov chain Monte Carlo settings
☐	☒	For hierarchical and complex designs, identification of the appropriate level for tests and full reporting of outcomes
☐	☒	Estimates of effect sizes (e.g. Cohen's *d*, Pearson's *r*), indicating how they were calculated

Our web collection on statistics for biologists contains articles on many of the points above.

Software and code

Policy information about availability of computer code

Data collection	FlowJo v12; Harmony v.5.0
Data analysis	GraphPad Prism v. 5.1; IDEAS v.6.2; NONMEM v.7.4; Pirana v.3.0; Perl-speaks-NONMEM v.4.8.1; Xpose 4.7.1, Las X.

For manuscripts utilizing custom algorithms or software that are central to the research but not yet described in published literature, software must be made available to editors and reviewers. We strongly encourage code deposition in a community repository (e.g. GitHub). See the Nature Portfolio guidelines for submitting code & software for further information.

Data

Policy information about availability of data

All manuscripts must include a data availability statement. This statement should provide the following information, where applicable:

- Accession codes, unique identifiers, or web links for publicly available datasets
- A description of any restrictions on data availability
- For clinical datasets or third party data, please ensure that the statement adheres to our policy

All data associated with this paper are present in the main manuscript or in the Supplementary Materials. The results from the primary in vitro screen of the ReFrame library generated in this study have been deposited in the Reframe database (https://reframedb.org/assays/A00279) under accession code A00279. The Phase I clinical study (ID: NCT02022306) described in this paper is deposited in the clinicaltrials.gov database (https://clinicaltrials.gov/ct2/results?cond=&term=NCT02022306&cntry=&state=&city=&dist=) under the accession code NCT02022306. Source data are provided with this paper.

Human research participants

Policy information about studies involving human research participants and Sex and Gender in Research.

Reporting on sex and gender	Sex: 98 males, 13 females, no gender reported
Population characteristics	Mean age: 38.9; Ethtnicity: Hispanic or latino 48/111, not Hispanic or latino 63/111; Race: White 74/111, Black/African American 34/110, Asian 1/111, Native Hawaiian or other 2/111.
Recruitment	Healthy, non-smoking subjects with no history of significant medical conditions, with a BMI included between 18 and 30 kg/m2, who weight at least 50 kg, between 18 and 60 years old, were selected for this study. Subjects were able and willing to give an informed consent. No bias was reported in the selection process.
Ethics oversight	Protocol submitted to IntegReview Institutional Review Board

Note that full information on the approval of the study protocol must also be provided in the manuscript.

Field-specific reporting

Please select the one below that is the best fit for your research. If you are not sure, read the appropriate sections before making your selection.

☒ Life sciences ☐ Behavioural & social sciences ☐ Ecological, evolutionary & environmental sciences

For a reference copy of the document with all sections, see nature.com/documents/nr-reporting-summary-flat.pdf

Life sciences study design

All studies must disclose on these points even when the disclosure is negative.

Sample size	Due to the exploratory nature of this study, no formal power or sample size calculations were used to determine cohort size. A sample size of 88 healthy subjects (8 subjects per cohort) for the SAD study and a sample size of 30 healthy subjects (10 per cohort) for the MAD study should provide adequate characterization of PK and safety assessments within this setting.
Data exclusions	Microsphiltration data were excluded when gametocytemia was higher than 10%
Replication	To ensure reproducibility dose-response analysis were repeated two times, post-screening validation of main hits 5 times (effect) and 4 times. All replications successfully confirmed the first observation.
Randomization	Subjects of the clinical study were randomly assigned to each cohort. Randomizations codes were assigned to the subjects.
Blinding	All study subjects, study investigators, and the Sponsor's staff involved in the conduct of the study were blinded to treatment assignment.

Reporting for specific materials, systems and methods

We require information from authors about some types of materials, experimental systems and methods used in many studies. Here, indicate whether each material, system or method listed is relevant to your study. If you are not sure if a list item applies to your research, read the appropriate section before selecting a response.

Materials & experimental systems

n/a	Involved in the study	
☒	☐	Antibodies
☐	☒	Eukaryotic cell lines
☒	☐	Palaeontology and archaeology
☒	☐	Animals and other organisms
☐	☒	Clinical data
☒	☐	Dual use research of concern

Methods

n/a	Involved in the study	
☒	☐	ChIP-seq
☐	☒	Flow cytometry
☒	☐	MRI-based neuroimaging

Eukaryotic cell lines

Policy information about cell lines and Sex and Gender in Research

Cell line source(s)	Plasmodium falciparum NF54 strain (BEI Resources, cat. no. MRA-1000) and transgenic modified 3D7 (BEI Resources, cat. no. MRA-102)
Authentication	Not done
Mycoplasma contamination	Not checked
Commonly misidentified lines (See ICLAC register)	No commonly misidentified lines

Clinical data

Policy information about clinical studies

All manuscripts should comply with the ICMJE guidelines for publication of clinical research and a completed CONSORT checklist must be included with all submissions.

Clinical trial registration	NCT02022306
Study protocol	The protocol of the study with all amendments has been made fully available in a separate file
Data collection	The study was conducted in the Healthcare Discoveries (a subsidiary of ICON Development Solutions) in San Antonio, Texas, USA.
Outcomes	Primary and secondary outcomes were pre-defined as follows: Safety and tolerability as a primary outcome, pharmacokinetics as a secondary outcome. Safety and tolerability were evaluated using standard measures, including adverse event (AE) monitoring, clinical laboratory tests (hematology, serum chemistry, coagulation, and urinalysis), vital signs, physical examinations, and 12-lead electrocardiograms (ECGs). Pharmacokinetics were evaluated by the collection of blood samples from each subject at following time points: predose (within 30 minutes before dosing, only in day 1 60 minutes before dosing), and 0.5,1,2,3,4,6,8 and 12 hours after dosing. Blood samples were analyzed to calculate the main PK parameters, such as Cmax, Tmax and t1/2.

Flow Cytometry

Plots

Confirm that:

☒ The axis labels state the marker and fluorochrome used (e.g. CD4-FITC).

☒ The axis scales are clearly visible. Include numbers along axes only for bottom left plot of group (a 'group' is an analysis of identical markers).

☒ All plots are contour plots with outliers or pseudocolor plots.

☒ A numerical value for number of cells or percentage (with statistics) is provided.

Methodology

Sample preparation	Plasmodium falciparum gametocytes in human erythrocytes were labelled with Hoechst staining. Ring-stage Plasmodium falciparum parasites were labelled with Sybr-Green
Instrument	FACSCAnto
Software	FlowJo v12
Cell population abundance	Gametocytes parasites were purified by density gradient before the flow cytometry analysis
Gating strategy	FSC-A and SSC-A to select red blood cells, FSC-A and FSC-H to exclude doublets, FITC to select GFP or Sybr-Green positive

☒ Tick this box to confirm that a figure exemplifying the gating strategy is provided in the Supplementary Information.
